# Immunologic response in patients with polytrauma

**DOI:** 10.1016/j.ncrna.2022.09.007

**Published:** 2022-09-21

**Authors:** Ural Mukhametov, Sergey Lyulin, Dmitry Borzunov, Tatiana Ilyasova, Ilgiz Gareev, Albert Sufianov

**Affiliations:** a Republican Clinical Hospital. G.G. Kuvatova, Ufa, 450071, Russian Federation; b Carmel Medical Center, Chelyabinsk, 454080, Russian Federation; c Ural State Medical University, Ekaterinburg, 620028, Russian Federation; d Рeoples’ Friendship University of Russia (RUDN University), 6 Miklukho-Maklaya Street, Moscow, 117198, Russian Federation; e Department of Neurosurgery, Sechenov First Moscow State Medical University (Sechenov University), Moscow, Russian Federation; f Bashkir State Medical University, Ufa, Russian Federation

**Keywords:** Immune response, Cytokines, Polytrauma, Biomarker, Infectious complications

## Abstract

**Purpose:**

It is now known that traumatic injury initiates a complex and dynamic immune response on the first day. It is believed that in patients with polytrauma, these immune responses contribute to the development of infectious complications. Therefore, understanding the immune response to trauma is critical to improving patient outcomes through the development of new therapies and improved resuscitation strategies. The purpose of this study is to examine the parameters of immunity in patients with severe polytrauma at the stages of surgical treatment (the nearest post-traumatic period and long-term periods) in the absence and presence of purulent-inflammatory complications.

**Methods:**

We retrospectively enrolled 188 patients after severely injured trauma and 210 control group at two Level-1 Trauma Centers. Peripheral blood was collected upon presentation to the hospital and at the following time points: 1, 3, 7, 14, 21, 30, 60 and 90 days, and daily during intensive care unit admission. T-lymphocytes analyses performed using a Beckman Coulter EPICS XL flow cytometer (USA) with monoclonal antibodies (Immunotech, France). Analyses of protein levels of cytokines/chemokines, immunoglobulins, and circulating immune complexes was using ELISA.

**Results:**

Under the influence of trauma, the content of T lymphocytes decreased due to the population of T-helpers. However, the number of B lymphocytes increased. The most pronounced activation of humoral immunity was observed by the 30th day of the post-traumatic period. Concentrations of interleukin-6 (IL-6), interleukin-8 (IL-8), tumor necrosis factor alpha (TNF-a), interleukin-10 (IL-10) on day 1 after injury were the highest. Later, in the post-traumatic period, a gradual decrease in the initially elevated cytokines was noted.

**Conclusions:**

As we continue to extrapolate new information on immune response factors associated with polytrauma, we will be better equipped to develop new therapeutic strategies to treat this serious clinical and social problem. In addition, individually adjusted immune control is an important interactive concept in polytrauma management.

## 1 Introduction

At present, trauma occupies one of the leading places among the causes of death among the young and most able-bodied part of the population. Polytrauma or severely injured trauma is a severely combined or multiple trauma, accompanied by an acute violation of vital functions of the body which requiring surgical treatment and intensive care measures in specialized multidisciplinary trauma centers [1,2]. It is in this group of victims that the highest mortality and disability is observed. Although the pathophysiological and immunological aspects of the pathogenesis of the course of polytrauma are being systematically studied, nevertheless, the features of the development of protective reactions in traumatic injuries require further in-depth research [3].

It is known that immunological changes in severe traumatic injuries cause an induced form of secondary immunodeficiency, accompanied by a predominant suppression of the functions of the cellular link of the immune system and nonspecific defense factors, characterized by changes in the number and activity of immunocompetent cells, as well as an imbalance in the cytokine/chemokines network of regulation of protective functions [4]. The incidence of infectious complications in polytrauma, according to various authors, ranges from 60 to 90% [1,2]. Moreover, among them, a high proportion is made up of severe infectious processes, including purulent-septic complications [5]. Thus, a consequence of polytrauma is a violation of the protective functions of the body. The study of the pathogenesis of polytrauma will allow, among other things, to predict the risk of development and generalization of infectious complications even before their clinical manifestation and to develop new methods of directed preventive and therapeutic immune correction. In polytrauma, the use of immunocorrection drugs can be focused on achieving two main targets. Firstly, the preventive use of immunoactive drugs provides non-specific immunoprophylaxis of infectious complications, in fact - the protection of the immune system from the adverse effects of acutely developing and accompanying trauma of immune dysfunctions. It is essential that the means of immunocorrection must be started in the acute period or the period of relative stabilization of the vital functions of the victims (no later than 24–36 h from the moment of injury). If drugs are not used, it is at this time that the foundation is laid for deep and diverse disorders of immunoreactivity, which will inevitably manifest themselves in the long term. Secondly, the use of immunoactive drugs to correct already developed immune disorders and concomitant to post-traumatic sepsis can be directed to pathogenetic therapy. The task of searching and developing possible approaches to the treatment of immune dysfunction and the prevention of infectious complications in polytrauma can be easily set on the basis of identifying only the manifestation of pathogenesis when an injury occurs [6]. Intensive development of this problem in recent cases of detection of its incidence and close connection with many aspects of general and molecular biology. In the late periods of injury, immunocorrection prevents the development of osteomyelitis and reduces the proportion of local complications of injury by 2.5 times. It has been proven that changes in hematological parameters during observations of patients with shin injuries determine the type of osteogenesis and act as adequate criteria for performing surgical osteosynthesis and immunocorrection [7]. This attracts to a wide range of specialists on the problems of the pathogenesis of polytrauma, which go far beyond the scope of theoretical medicine. In this study, we to examine the parameters of immune response in patients with polytrauma at the stages of surgical treatment (the nearest post-traumatic period and long-term periods) in the absence and presence of purulent-inflammatory complications.

## 2 Material and methods

### 2.1 Patients and control group

This retrospective study included 188 polytrauma patients who admitted to the intensive care unit (ICU) of a two Level-1 Trauma Centers from June 2019 to August 2021. Participating clinical facilities included Ilizarov Center (Kurgan, Russia) and Republican Clinical Hospital G.G. Kuvatova (Ufa, Russia). Patients were divided into 2 groups: 112 polytrauma patients with a favorable course at the stages of surgical treatment (without complications) and 76 polytrauma patients with purulent-inflammatory complications including 33 pneumonia cases. A patient with AIS≥3 in two or more different body regions was considered to have polytrauma. All patients with an overall ISS>15 and hospitalized for treatment of traumatic injuries were included in the study. Exclusion criteria were presented to our institution greater than 24 h after injury, pre-existing immunologic dysfunction, history of organ failure, and pregnancy. The immunological parameters of 210 relatively healthy volunteers were used as a control group. 210 healthy volunteers were grouped by similar age and sex. The study did not include patients with concomitant somatic pathology that could affect the results of an immunological study (carriers of hepatitis C virus (HCV), human immunodeficiency viruses (HIV), hepatitis B surface antigen (HBsAg), patients with autoimmune diseases). The study was approved by our respective institutions’ institutional review boards prior to any patient data collection. Written informed consent was obtained from all subjects. Clinical characteristics of polytrauma patients are summarized in Table 1 and Table 2.

**Table 1:** Polytrauma cohort baseline characteristics of enrolled patients.

Characteristics	Patients (n = 188)
Median age, years	31 (IQR 23–55)
Sex	
Male, n	122
Female, n	66
Positive BAL, n	48
Median ISS	20
Purulent-inflammatory complications, n	76
Pneumonia, n	33
Mechanism of trauma, n	
Road transport incidents	80
Falls from heights	39
Falling objects	35
Sports	16
Сriminal	8

**Abbreviations:** BAL, blood alcohol level; IQR, interquartile range; ISS, illness severity score.

**Table 2:** Clinical parameters of polytrauma patients with complications.

Characteristics	Purulent-inflammatory complications (n = 76)	Pneumonia (n = 33)
Median age, years	31.0 ± 3.63	31.0 ± 4,57
Male, n	44	11
Female, n	32	22
Damage localization		
LBF + TBI +CI	13	4
LBF + TBI + CI + spinal injury	2	2
LBF + TBI +CI + pelvic bone injury	5	1
LBF + TBI +CI + abdominal injury	6	12
LBF + TBI + spinal injury + pelvic bone injury	26	0
LBF +CI + spinal injury	7	6
LBF +CI + spinal injury	4	3
LBF +CI + pelvic bone injury	13	5

LBF, Long bone fractures; TBI, Traumatic brain injury; CI, Chest injury.

### 2.2 Sample preparation

The blood samples of patients with polytrauma was collected upon presentation to the hospital and at the following time points: 1, 3, 7, 14, 21, 30, 60 and 90 days, and daily during intensive care unit admission. Blood samples including control group were all processed within 2 h of collection. Whole blood had been stored at 4–8 °C before the serum or plasma is separated, but not frozen it. Blood samples were collected in two tubes. Blood samples without anticoagulants for obtaining serum before determining the content of cytokines/chemokines and immunoglobulins in them. At all plasma samples were extracted from ethylenediaminetetraacetic acid (EDTA) tubes for the subsequent isolation of immune cells. The plasma and serum was aliquoted into separate cryovial tubes and immediately frozen at −80C.

Delineation of human peripheral blood lymphocytes was carried out by Beckman Coulter EPICS XL flow cytometer (USA) using monoclonal antibodies from (Immunitech, France). The quantitative determination of immunoglobulins, cytokines/chemokines, and circulating immune complexes (CIC) was carried out by ELISA on a microplate absorbance reader ELX 808 (BIO-TEK® Instruments Inc., Winooski, VT, USA) using reagent kits produced by ZAO Vector-Best (Russia). For investigated the phagocytic activity of neutrophils (phagocytic index and phagocytic number) a standard technique was used, based on the quantitative determination of the absorption and digestion capacity of neutrophils in relation to a microbial culture (S. epidermidis strains, No. 9198 “NIIEM” SZO RAMS). Blood smears stained according to the Romanowsky-Giemsa method were used to count neutrophil extracellular traps (NETs). The percentage of neutrophils that passed the stage of nuclear transformation and released free chromatin into the extracellular space in the form of network-like structures was calculated.

### 2.3 Statistical analysis

Analysis of the results of the study was carried out using the AtteStat 12.0.5 software, made as an add-on to Microsoft Excel of the Microsoft Office software product. The data obtained were processed using non-parametric statistics methods using the Mann-Whitney-Wilcoxon (MWW) *U* test. The study results are presented as median and interquartile range (IQR) (25th and 75th percentiles). A P value of <0.05 (*) or < 0.01 (**) was considered statistically significant.

## 3 Results

### 3.1 Analysis of indicators of cellular and humoral immunity

In this study was demonstrated under the influence of traumatic injury the content of T lymphocytes decreased due to the population of CD4^+^ T helper (Th) lymphocytes (Table 3). On the 3rd day of the post-traumatic period, the content of T lymphocytes practically did not change. At the same time, there were changes in the subpopulation composition of lymphocytes, where the number of Th increased against the background of a slight decrease in the number of CD8^+^ сytotoxic T lymphocytes (CTLs). By the 7th day, there was a tendency to an increase in the number of the general population of T lymphocytes. By the 14th day after the injury, the number of the total population of T lymphocytes approached the values of the control group. In the long term (on day 90), the main indicators of cellular immunity did not have statistically significant differences from those of the control group. Up to 21 days of the post-traumatic period, increased values of cells carrying markers of lymphocyte activation persisted. By the 30th day, lymphocytes from the markers of early activation began to decrease and by the 60th day they reached the values of the control group. T lymphocytes carrying markers of late activation remained high for 90 days. T lymphocyte activation markers in patients with polytrauma are demonstrated in Table 4.

**Table 3:** Parameters of cellular immunity in patients after severely injured trauma at the stages of treatment.

Period	СD3^+^СD19^-^%	СD3^+^СD4^+^ %	СD3^+^СD8^+^ %
1 day	71.4^+^ (58.0–76.0)	42.0^+^ (27.1–43.1)	26.1 (18.7–31.2)
3 day	72.9^+^ (58.7–79.6)	47.8* (39.0–51.2)	22.8^+^ (17.1–30.2)
7 day	73.5* (68.0–83.0)	46.7* (40.8–53.6)	25.1 (17.8–31.4)
14 day	75.6* (73.1–82.2)	49.6* (42.0–55.2)	24.4 (19.1–30.6)
21 day	80.6* (71.8–85.0)	48.7* (43.3–56.0)	25.3 (21.3–33.3)
30 day	76.0* (71.5–81.3)	47.1** (44.8–55.1)	28.3 (21.4–31.4)
60 day	81.2^+^* (75.8–85.1)	44.5* (42.0–47.8)	33.3^+^* (26.0–39.0)
90 day	81.7* (70.8–83.9)	44.7* (36.3–50.0)	31.8* (25.0–38.2)
Control group	76.0 (73.4–78.7)	49.8 (41.0–53.3)	25.2 (23.3–33.5)

**Note:** * (р ≤ 0.05) and ** (р ≤ 0.01), in comparison with indicators of 1 day; + (р ≤ 0.05), in comparison with the indicators of the control group.

**Table 4:** T lymphocyte activation markers in patients after severely injured trauma at the stages of treatment.

Period	СD3^+^ СD25^+^ %	СD3^+^HLADR %
1 day	9.7^+^ (7.4–14.0)	6.4^+^ (4.1–9.2)
3 day	6.9^+^ (6.3–11.3)	5.6^+^ (4.1–7.7)
7 day	9.8^+^ (7.8–11.9)	5.5^+^ (4.0–8.0)
14 day	8.4^+^ (6.3–14.0)	7.1^+^ (5.2–10.1)
21 day	9.1^+^ (8.1–10.5)	9.2^+^ (5.8–13.0)
30 day	7.4^+^* (5.5–10.2)	8.2^+^ (5.0–17.5)
60 day	4.5^+^* (4.0–6.6)	11.0^+^ (5.7–15.0)
90 day	4.0* (3.8–4.4)	8.4^+^ (5.1–9.2)
Control group	3.2 (2.7–4.3)	3.2 (2.1–4.0)

**Note:** * (р ≤ 0.05), in comparison with indicators of 1 day; + (р ≤ 0.05), in comparison with the indicators of the control group.

**Fig. 1:**
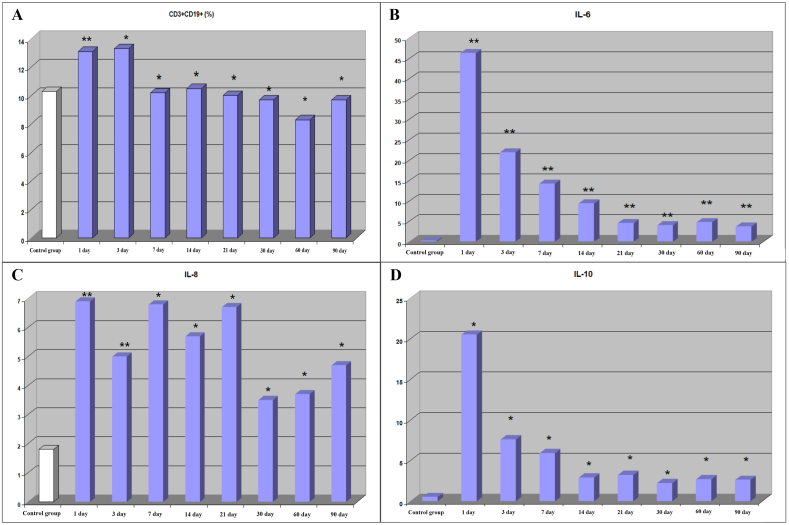
The level of B lymphocytes **(A)** and cytokines/chemokines **(B**–**D)** in patients after severely injured trauma at the stages of treatment. Statistical significance (*p ≤ 0.05 and **p ≤ 0.01). Note: Only the indicators of the 1 day were compared with those of the control group. The indicators of the rest days were compared with the indicators of 1 day.

Under the influence of traumatic injury, the humoral immune response was activated. In comparison with the control group, the number of B lymphocytes, immunoglobulins and CIC increased (Fig. 1A, Table 5). On the 3rd day, a short-term drop in the concentration of IgM and IgG was revealed, due to the reaction of the immune system to surgical intervention against the background of an immune imbalance caused by polytrauma. Starting from the 7th day, an increase in CIC and IgE was noted. The most pronounced activation of humoral immunity was observed by the 30th day of the post-traumatic period. By day 60, the level of CIC returned to normal. By day 90, a moderate increase in the concentrations of IgA, IgG, and IgE remained.

**Table 5:** Indicators of the concentration of IgA, IgM, IgG, IgE, and сcirculating immune complexes (CICs) in patients after severely injured trauma at the stages of treatment.

Period	IgA (mg/ml)	IgM (mg/ml)	IgG (mg/ml)	IgE (mg/ml)	CICs
1 day	2.0^+^ (1.0–2.5)	1.4^+^ (0.9–2.6)	10.8^+^ (7.9–16.0)	29,0^+^ (9,4–49,0)	22,0^-^(15,5–56,0)
3 day	1.8^+^ (0.9–3.2)	0.77^+^* (0.63–1.1)	7.8* (6.2–15.0)	33,0^+^ (8,0–55,4)	32,0^-^(12,0–84,0)
7 day	2.2^+^ (1.4–3.6)	1.32^-^(0.7–1.6)	11.3^-^(7.8–17.8)	61,0 ^++^* (22,0–219,0)	53,0* (18,0–78,0)
14 day	2.4^+^* (1.9–4.0)	1.5^-^(0.9–2.4)	14.0* + (9.9–20.7)	49,0^++^* (12,0–236,0)	55,0* (22,0–94,0)
21 day	1.9^+^ (1.21–3.1)	1.35^-^(1.03–1.9)	12.0^+^ (8.6–16.9)	46,0^++^* (11,0–184,0)	65,0^+^* (49,0–104,0)
30 day	2.9^+^ (1.2–4.0)	1.8^+^* (1.3–3.3)	18.9 ^+^* (11.0–24.11)	24,0 ^++^* (11,0–107,0)	47,5* (27,3–69,0)
60 day	1.93^+^ (1.3–3.5)	1.5^-^(0.9–2.4)	14.7^+^ (9.9–18.4)	22,7^+^ (9,76–46,0)	45,0^-^(29,0–58,0)
90 day	2.0^+^ (1.9–3.0)	1.16^-^(0.8–1.7)	13.6^+^ (9.2–15.3)	20,0^+^ (13,0–43,0)	45,0^-^(26,5–72,0)
Control group	1.14 (0.75–2.0)	1.26 (0.63–2.24)	7.1 (5.6–14.7)	12,6 (3,75–21,0)	45,0 (29,0–53,0)

**Note:** * (р ≤ 0.05), in comparison with the indicators of the preoperative period; + (р ≤ 0.05) and ++ (р ≤ 0.01), in comparison with the indicators of the control group; -, not statistically significant.

### 3.2 Cytokines/chemokines profile and innate immunity

To obtain a systematic picture of inflammatory processes in polytrauma, it is necessary to simultaneously study a large number of cytokines. Levels of certain cytokines, such as interleukins, can be used to predict the correlation of infectious complications in patients with polytrauma. The concentrations of interleukin 6 (IL-6), interleukin 8 (IL-8), interleukin 10 (IL-10), and tumor necrosis factor alpha (TNF-a), on day 1 after traumatic injury were increased (Fig. 1B–D). Moreover, the TNF-a concentration was increased only on the 1st day (6.4 (4.1–9.2), p < 0.05) of 90 days of the entire post-traumatic period. In the post-traumatic period, a gradual decrease in initially elevated cytokines/chemokines was noted. At the same time, only TNF-a reached the level of the control group by day 90. From 3 days to 60 days after the injury, a decrease in the number of natural killer (NK) cells was observed, which indicated violations in the innate immunity system (Table 6).

**Table 6:** Indicators of innate immunity in patients after severely injured trauma.

Period	СD3^−^СD16^+^ СD56^+^ %	СD14^+^HLA-DR %
1 day	14.1^-^(7.5–21.9)	51.1^+^ (40.9–61.9)
3 day	8.1* (4.5–20.3)	57.3^+^ (41.9–76.8)
7 day	7.4^+^* (4.6–12.1)	61.1^+^* (44.4–71.5)
14 day	9.5* (5.5–13.6)	66.4^+^* (56.2–77.3)
21 day	7.3* (5.6–14.8)	74.9^+^* (61.9–81.3)
30 day	10.5* (6.2–13.0)	76.6^+^* (73.3–81.3)
60 day	7.8* (6.2–11.6)	76.2^+^* (70.3–85.6)
90 day	10.0^-^(7.6–19.0)	75.6*^+^ (70.4–85.3)
Control group	10.95 (8.58–14.3)	91.6 (88.1–93.4)

**Note:** * (р ≤ 0.05), in comparison with indicators of 1 day; + (р ≤ 0.05), in comparison with the indicators of the control group; -, not statistically significant.

By the 90th day, the indicator reached the values of the control group. The pronounced changes in the monocytes and macrophages were evidenced by a pronounced decrease in expression of human leukocyte antigen class II (HLA-DR) on monocytes. An increase in the indicator was noted starting from the 7th day of the post-traumatic period. At the same time, the indicator did not rise to the values of the control group even 90 days after the traumatic injury. Similar changes occurred with neutrophils (Table 7). From day 1 to day 14 inclusive of the post-traumatic period, a decrease in the absorption capacity of neutrophils was observed. Against the background of a reduced effector function, activation of oxygen-dependent killing activity of neutrophils was noted (a significant increase in nitroblue tetrazolium (NBT) test, both spontaneous and stimulated). The values phagocytic index and phagocytic number on day 21 exceeded the values of 1 day. By the 90th day of the post-traumatic period, the phagocytic index reached the values of the control group; there was a tendency to normalization of the phagocytic number, and the NBT test, while they did not reach the values of the control group.

**Table 7:** Indicators of phagocytic activity of neutrophils in patients after severely injured trauma.

Period	Phagocytic index %	Phagocytic number %	Spontaneous NBT test %	Stimulated NBT test %
1 day	67.0 (62.2–71.1)	5.42^+^ (4.25–6.1)	12.2^++^ (10.0–15.5)	81.0^+^ (65.25–89.25^+)^
3 day	56.0 + (53.0–71.0)	4.0*^+^ (3.5–4.5)	16.0*^++^ (14.0–19.5)	82.0^+^ (76.0–85.5)
7 day	78.0 (69.0–79.5)	5.0*^+^ (4.5–5.0)	20.0*^++^ (16.5–23.0)	90.0*^++^ (86.0–92.0)
14 day	76.0 (68.0–78.0)	5.0*^+^ (4.5–5.0)	11.0*^+^ (9.0–11.5)	82.0^+^ (81.0–83.0)
21 day	87.0* (81.5–88.5)	8.0* (7.0–8.5)	11.0^+^ (10.5–11.5)	83.0^+^ (77.5–84.5)
30 day	76.0*^+^ (74.0–82.0)	5.0*^+^ (5.0–5.5)	12.0^+^ (10.5–12.5)	86.0^+^ (78.0–87.5)
60 day	73.0*^+^ (70.0–79.0)	5.0^+^ (5.0–6.0)	10.0*^+^ (8.5–10.5)	76.0^+^ (74.5–80.0)
90 day	71.0 (69.5–74.5)	6.0^+^ (5.5–6.0)	8.0*^+^ (7.5–9.0)	66.0^+^ (63.5–69.0)
Control group	71.0 (68.5–78.0)	7.3 (6.8–8.0)	6.0 (4.5 ÷ 7.0)	57.0 (48.0 ÷ 62.3)

**Note:** * (р ≤ 0.05), in comparison with indicators of 1 day; + (р ≤ 0.05) and ++ (р ≤ 0.01), in comparison with the indicators of the control group; NBT, nitroblue tetrazolium.

### 3.3 Dynamics of immunity indices in polytrauma patients with a favorable course and purulent-inflammatory complications in the post-traumatic period

**Fig. 2:**
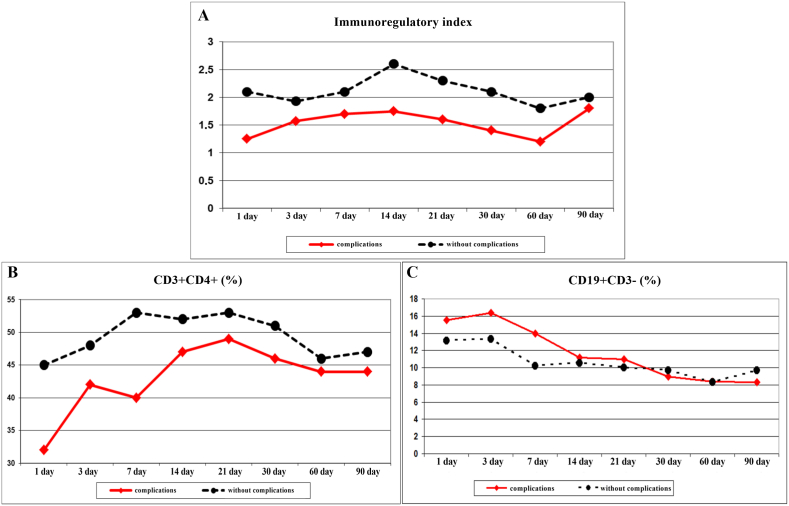
Immunoregulatory index **(A)**, the circulating level of T helpers (Th) **(B)** and in B lymphocytes **(C)** in patients after severely injured trauma at the stages of treatment with a favorable course of the post-traumatic period and the development of complications.

**Fig. 3:**
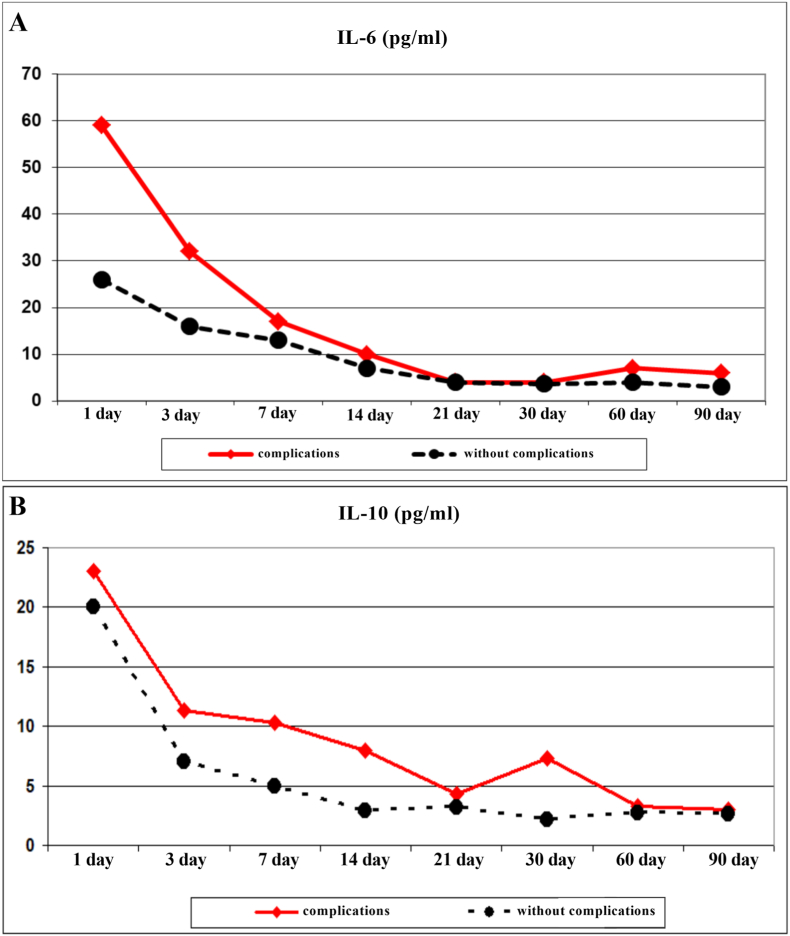
The circulating level of interleukin 6 (IL-6) **(A)** and interleukin 10 (IL-10) **(B)** in patients after severely injured trauma at the stages of treatment with a favorable course of the post-traumatic period and the development of complications.

In the patients of the development of purulent-inflammatory complications during the next 30 days of the post-traumatic period, the dynamics of immunity indicators changed. The immunoregulatory index, in the polytrauma patients with purulent-inflammatory complications, was lower than in the patients without purulent-inflammatory complications due to the reduced values of Th (Fig. 2A and B). The level of Th in the patients with complications approached the values of the patients without complications only by day 90 of the post-traumatic period. At all other periods, the level of Th was lower than in the patients without complications. The level of B lymphocytes in the patients with complications exceeded the values of the patients without complications up to 14 days of the post-traumatic period (Fig. 2C). In additional, in the immediate post-traumatic period, the level of IL-6 was higher than in the patients without complications (Fig. 3A). IL-10 for 30 days of the post-traumatic period in the patients with complications significantly exceeded the values of the patients with a favorable course of the post-traumatic period (Fig. 3B).

**Fig. 4:**
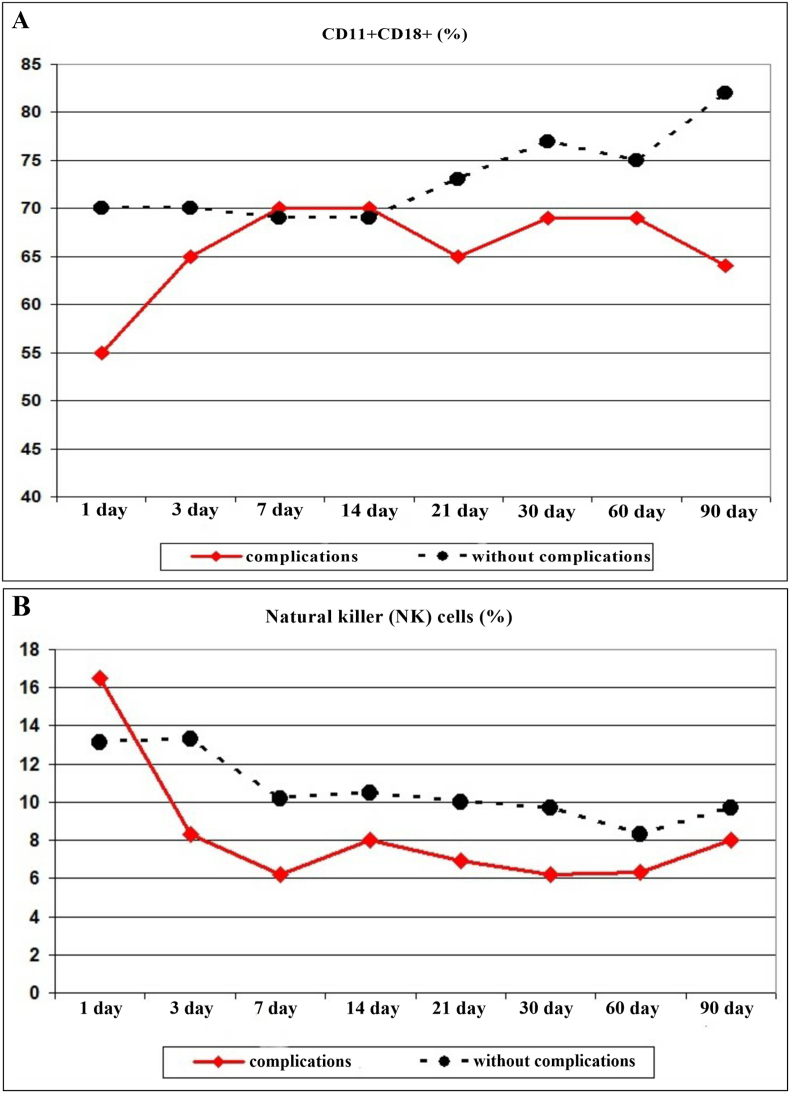
The circulating level of CD11 + CD18^+^**(A)** and natural killer (NK) cells **(B)** in patients after severely injured trauma at the stages of treatment with a favorable course of the post-traumatic period and the development of complications.

**Fig. 5:**
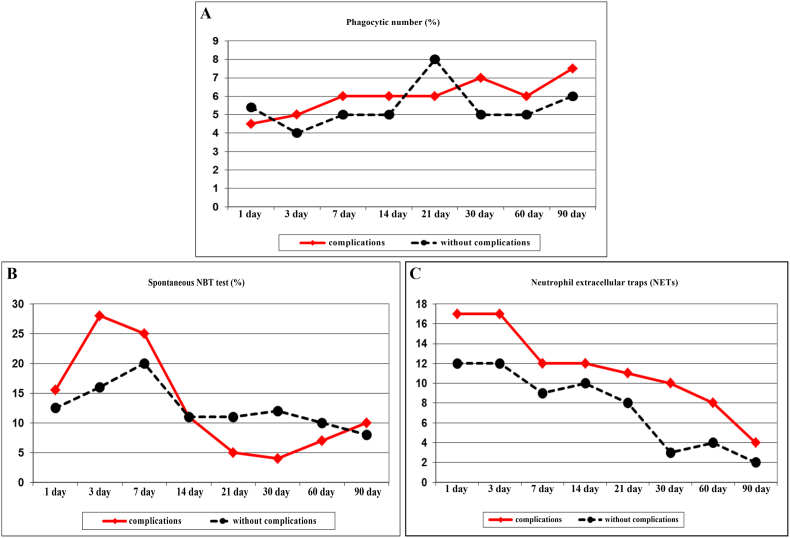
The value of a phagocytic number **(A),** spontaneous nitroblue tetrazolium (NBT) test **(B),** and neutrophil extracellular traps (NETs) **(C)** in patients after severely injured trauma at the stages of treatment with a favorable course of the post-traumatic period and the development of complications.

Changes in the indices of innate immunity were also observed. In the patients with complications, the adhesive ability of neutrophils was significantly reduced (Fig. 4A). Starting from day 3, the level of NK cells decreased in the patients with complications, remaining below the indices of the patients with a favorable post-traumatic period during all subsequent stages (Fig. 4B). During the main periods, starting from 3 days, the phagocytic number in the patients with complications exceeded the indicators in the patients without complications (Fig. 5A). The spontaneous NBT test in the short term after the traumatic injury in the patients with complications exceeded the values in the patients without complications. From the 21st day, the dynamics changed. From 30 to 60 days of the post-traumatic period, the oxygen-dependent killing activity of neutrophils in the patients with complications sharply decreased, which can be explained by the disruption of adaptive-compensatory mechanisms (Fig. 5B). There was a significant increase in the number of NETs in patients with complications both in the immediate post-traumatic period and at subsequent traumatic injury stages (Fig. 5C). At the same time, an increase in the number of NETs in the patients with complications occurred due to mature forms characteristic of the inflammatory process.

#### 3.3.1 Innate immunity and pneumonia

In this study, indices of innate immunity were studied separately in patients with polytrauma in the inductive phase of the immune response during the development of pneumonia. In the patients with pneumonia, the next day after the traumatic injury, there was a more pronounced leukocytosis than in the patients without complications (Table 8). The level of leukocytes increased due to the populations of monocytes and neutrophils.

**Table 8:** Indicators of innate immunity in patients after severely injured trauma with including purulent-inflammatory complications.

Indicators	No complications	Pneumonia	Control group
Leukocytes, 10^9^/L	10.4*^+^ (8.4–12.2)	12.6 ^++^ 10.0–18.6	5.6 (3.2–8.4)
Monocytes, 10^9^/L	1.01*^+^ (0.85–1.21)	0.78^+^ 0.63–0.9	0.39 (0.22–0.5)
Neutrophils, 10^9^/L	7.63*^+^ (6.5–8.54)	10.6^++^ 8.2–12.8	3.28 (2.9–4.1)
Average cytochemical coefficient (ACC)	2.6^+^ (2.5–2.8)	2.6^+^ 2.55–2.85	2.2 (2.0–2.4)
Spontaneous NBT test, %	12.2^+^ (10.0–15.5)	14.0^+^ 12.0–23.0	6.0 (4.5 ÷ 7.0)
Stimulated NBT test, %	81.0^+^ (65.25–89.25)	80.5^+^ (73.75–86.25)	54.0 (52.0–60.0)
Phagocytic index, %	67.0 (62.2–71.1)	61.4^+^ (51.2–66.3)	71.0 (64.9–75.0)
Phagocytic number, %	5.42^+^ (4.25–6.1)	5.0^+^ (4.6–6.2)	7.3 (6.2–7.7)
Number of active phagocytes (NAP), 10^9^/L	4.78*^+^ (3.2–6.5)	6.3^++^ (4.2–8.5)	2.3 (1.9–2.5)
Absolute phagocytic index (API),10^9^/L	38.8*^+^ (37.5–52.2)	53.5^+^ (45.2–79.5)	24.0 (20.0–35.5)

**Note:** * (р ≤ 0.05), in comparison with the indicators of the patients group with pneumonia; + (р ≤ 0.05) and ++ (р ≤ 0.01), in comparison with the indicators of the control group; NBT, nitroblue tetrazolium.

**Fig. 6:**
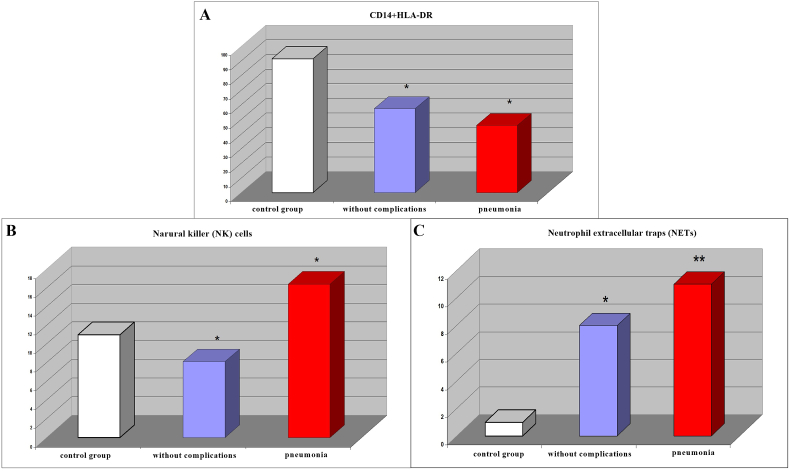
The circulating level of CD14+HLA-DR **(A)** and natural killer (NK) cells **(B)**, and the value of neutrophil extracellular traps (NETs) **(C)** in patients after severely injured trauma at the stages of treatment with a favorable course of the post-traumatic period and the development of pneumonia. Statistical significance (*p ≤ 0.05 and **p ≤ 0.01). Note: The indicators of patients without complications and with pneumonia were compared with the control group.

Against the background of monocytosis, a decrease in the level of CD14 + HLA-DR was recorded (Fig. 6A). According to the results of our study, the level of NK cells in the patients with pneumonia significantly exceeded the values in the patients without complications, which can be explained by the massive influx of bacterial antigens into the bloodstream (Fig. 6B). At the same time, the absolute values of the indicator in both groups with polytrauma were significantly lower than those of the control group. With regard to NETs in patients with polytrauma, we found that the NETs level in the patients without complications was significantly lower than in the patients with pneumonia (Fig. 6C). In the control group, the early stages of NETs were not detected, and mature forms represented single NETs.

## 4 Discussion

Polytrauma continues to be in the spotlight researchers, as it is one of the most common causes of mortality and disability in victims One of the most important directions in the development of modern medicine is the development of methods for diagnosing and prognosis the course of diseases based on determining the degree of impairment of functional activity and the possibility of correcting the protective functions of the body [8]. The peculiarity of these studies at the present stage is, on the one hand, in an in-depth study of the cellular and molecular mechanisms of innate and adaptive immunity and their regulation, and on the other, in obtaining fundamentally new important information based on the analysis of the functional activity of immunocompetent cells, which largely determines the potential protective functions [[9], [10], [11]].

Our study results allow us to conclude that an imbalance of the immune system occurs after severely injured trauma at various times of the post-traumatic period. It has been established that the functional state of the cellular and humoral immunity in patients with polytrauma was characterized by a phasic flow, depending on the time elapsed since the moment of traumatic injury. At the beginning of the early period of traumatic injury was the suppression of the circulating level T lymphocytes. The decrease in the total level of T lymphocytes, as can be seen from the data, was mainly due to Th and NK cells. NK cells play an important role in the pathogenesis of traumatic injury, participating in inflammatory reactions through killing, as well as the synthesis of cytokines that activate new participants in the immune defense [12]. According to the literature, a decrease in the population of NK cells is usually observed in the near term after a severe trauma against the background of lymphopenia [13,14]. Pronounced early changes in the level of NK cells in polytrauma patients associated with the development of complications [12]. In addition, the B lymphocyte count was high in the early stages of the post-traumatic period. Such an increase in the level of B lymphocytes testified to the activation and the initial process of antitelogenesis.

Acute circulatory disorders in the extremities in polytrauma and are mainly associated with direct mechanical detection or the presence of acute compartment syndrome [15]. It is known that T lymphocytes play an important role in the restoration of ischemic tissue areas [16]. At the same time, some authors believe that CD8+T cells can be the main “conductors” of the processes of inflammation, angiogenesis, and osteogenesis, since they infiltrate the site of damage before the migration of monocytes (macrophages) [16]. Vascular endothelial growth factor (VEGF)-mediated neovascularization is initiated primarily by CD4^+^ T cells, while CD8^+^ T cells induce recruitment of CD4^+^ T cells by interleukin-16 (IL-16) [17]. VEGF has been shown to induce type 1 T helper (Th1) cells polarization of T cells *in vitro* [18]. It is also worth noting that T cells play an important role in polarizing monocytes into a pro-angiogenic phenotype through intercellular contact and paracrine signaling mechanisms. Perhaps one of the reasons for the decrease in the number of circulating T-lymphocytes is associated with compensatory reactions of the body during polytrauma, namely, their movement to areas of damaged ischemic tissue.

After removing a patient with trauma from the acute period, there is a risk of infectious complications up to the development of sepsis, which is one of the most important causes of death of patients [19]. The most common post-traumatic complications of an infectious nature are also pneumonia, peritonitis, and wound infection [19]. The increase in the circulating level of the cytokines/chemokines was at the beginning of the early period of traumatic injury and with the development of purulent-inflammatory complications. The imbalance of cytokines/chemokines indicated a violation of the central mechanisms of immunoregulation and the transition of the cells of the immune system, which synthesize these cytokines/chemokines, to the level of self-regulation [20]. According to our results, during the first days after polytrauma, there was a significant decrease in the expression of HLA-DR on monocytes, more pronounced in the patients with pneumonia. This indicator is one of the highly informative prognostic markers of infectious complications, multiple organ failure, and death. The possibility of using the indicator to identify anti-inflammatory immunological phases is not excluded [21,22].

The most numerous leukocytes found in injured tissues are neutrophilic granulocytes (NG), which belong to the most mobile population of innate immunity cells, which play an important role in anti-infectious protection [23]. In the patients with pneumonia, we found a decrease in a phagocytic index and phagocytic number. At the same time, the values of phagocytic parameters (number of active phagocytes (NAP) and absolute phagocytic index (API)) in both patients groups significantly exceeded the values of the control group, where the changes were more pronounced in the patients with pneumonia. Unfortunately, it is difficult to say the reason for the decrease in the phagocytic index in polytrauma. However, the possible reason for the decrease in the phagocytic index may be due to reduced production of phagocytes, their rapid decay, impaired mobility, impaired absorption of a foreign agent, impaired processes of its destruction, etc. All this indicates a decrease in the body's resistance to generalized infections, in particular those causing pneumonia [24,25]. As you know, the peak of activity of polymorphonuclear neutrophils (PMNs), which begin to actively migrate to the foci of inflammation, occurs 3–4 h after traumatic injury [26]. Further, there is a decrease in phagocytic activity of neutrophils and the addition of infection, even more, suppresses the antibacterial activity of neutrophils, which is accompanied by a decrease in the phagocytic index and phagocytic number. Along with this, the flow of immature forms of NG into the bloodstream increases [23]. Despite the predominance of immature forms of NG, which do not yet sufficiently possess all the properties of mature cells, neutrophils increase the production of reactive oxygen species (ROS) during this period [27].

In both patient groups, activation of oxygen-dependent metabolism of NG was observed. An increase in the values of both spontaneous and stimulated NBT tests was found. In both patient groups, there was an increase in the oxygen-dependent lysosomal killing activity of neutrophils, which was accompanied by an increase myeloperoxidase level [28]. Neutrophils can perform their bactericidal and cytotoxic functions by means of classical phagocytosis, but extracellular (external) killing is also no less important for maintaining homeostasis [29]. Extracellular killing is the recently discovered ability of neutrophils to form reticular structures by releasing DNA strands into the extracellular space. It is known that the intensity of NETs formation directly depends on the activity of the infectious process and oxygen-dependent metabolism of neutrophils. Massive traumatic tissue injury is a factor contributing to the activation of extracellular killing [21,30]. With regard to NETs in polytrauma patients, we found that the content of NETs in the uncomplicated course of the post-traumatic disease was significantly lower than in the group with pneumonia. In polytrauma patients with a favorable course in the post-traumatic period, the more active NETs formation was noted at the initial stage of netosis. In the patients with pneumonia, mature forms of NETs prevailed, which was explained by the stimulating effect of pathogenic microflora against the background of increasing oxygen-dependent metabolic activity of neutrophils.

Existing studies show that surgical injury and blood loss cause marked suppression of cellular immunity and increased susceptibility of patients to infections [31,32]. In particular, the interactions of monocytes and T cells change after injury and blood loss. Thus, immunomodulatory therapy in surgical patients should protect lymphocytes, macrophages, granulocytes, and endothelial cells from both hyperactivation and depletion. This opinion is supported by the results of clinical studies aimed at the inhibition of specific mediators, i.e. interleukin-1 (IL-1) or TNF-α [33]. Despite promising results in experimental settings, these trials have failed to improve results in clinical practice. Therefore, the further direction of this study is a more specific study of changes in the immune system in various cohorts of patients with polytrauma, including groups with and without surgical treatment.

The role of the immune system in the nature of the defense response to severe traumatic injury is constantly being studied, new mechanisms for the development of complications are being discovered, immunological diagnostics are improving, and new effective immunotropic drugs appear. However, the high incidence of complications and deaths occurring in the early period in polytrauma patients testifies to insufficient knowledge of the mechanisms of immunological protection, insufficient development, and application of immunological criteria for the severity of the condition, indications, and contraindications for performing treatment including immune correction. To develop criteria and methods for prognosis the risk of developing and generalizing infectious complications, as well as to improve targeted prevention and immunocorrection in patients with polytrauma, it is necessary to study in more detail the peculiarities of disorders of the immune system in the early period after traumatic injury.

## 5 Conclusions

The results of this study make it possible to assess some mechanisms of violations of the protective functions in polytrauma and open up the possibility of prognosis the course of the traumatic injury and its targeted immune correction.

## Author contributions

Investigation, Validation, Analysis and interpretation of data: Sergey Lyulin and Dmitry Borzunov; Conceptualization and design of study, Writing - original draft, review & editing: Ural Mukhametov, Tatiana Ilyasova and Ilgiz Gareev; Acquisition of data: Albert Sufianov; Funding acquisition: Albert Sufianov; Supervision: Sergey Lyulin. Approved the final version of the manuscript on behalf of all authors: Sergey Lyulin.

## Data availability statement

The original contributions presented in the study are included in the article, further inquiries can be directed to the corresponding author/s.

## Ethics approval and consent to participate

The studies involving human participants were reviewed and approved by the Ethics Committee of by the Ilizarov Center (Kurgan, Russia) and Republican Clinical Hospital G.G. Kuvatova (Ufa, Russia).

## Funding

This study was supported by the Bashkir State Medical University Strategic Academic Leadership program (PRIORITY-2030).

## Declaration of competing interest

The authors declare no conflict of interest.
